# Reinforcement of Silty Soil via Regenerated Fiber Polymer: A Study on Microscopic Mechanisms

**DOI:** 10.3390/ma16206741

**Published:** 2023-10-18

**Authors:** Xiaoyan Liu, Shihao Yan, Lulu Liu

**Affiliations:** 1 School of Mechanics and Civil Engineering, China University of Mining and Technology, Xuzhou 221116, China; luckyliuxiaoyan@cumt.edu.cn; 2 School of Civil Engineering, Dalian University of Technology, Dalian 116024, China; ysh12006010@mail.dlut.edu.cn; 3 State Key Laboratory for Geomechanics and Deep Underground Engineering, China University of Mining and Technology, Xuzhou 221116, China

**Keywords:** silty soil, regenerated fiber polymer, mechanics, microscopic mechanisms

## Abstract

Utilizing regenerated polyester fibers (RPFs) for the reinforcement of silty road bases not only enhances the soil’s engineering performance but also offers a sustainable method for repurposing waste polyester bottles. To investigate the engineering properties and microscopic behaviors of this reinforced silty soil, a series of extensive physico-mechanical tests were conducted, supplemented by Scanning Electron Microscopy (SEM) analyses. These evaluations focused on the influence of variables such as fiber content, fiber length, moisture content, and curing duration on the modified soil’s performance. The fiber content of the test was 0–1%, and the fiber length was 6–17 mm. The results indicate that curing age had a less significant impact on liquid and plastic limits than the addition of fiber, along with a marginal decline in the plasticity index over time. The rate of shrinkage in the unmodified soil was between 1.04 and 1.45 times higher than that in the fiber-reinforced soil, indicating effective shrinkage control by the fibers. However, variations in maximum dry density (*ρ*_dmax_) were insignificant across different fiber contents, while a slight increase was observed in the optimum moisture content (OMC) as fiber dosage increased. After a 28-day curing period, the resilient modulus and California Bearing Ratio (CBR) met highway road base design standards. A decline in unconfined compressive strength was noted when the fiber dosage exceeded 0.2%. The addition of fibers mitigated diagonal cracking and shifted the failure pattern towards a more ductile mode. This research contributes scientific insights for the broader application and promotion of silty road base improvement techniques using RPFs.

## 1. Introduction

Encountering silty road bases during transportation infrastructure development is common. These soils are inherently difficult to compact and exhibit poor engineering characteristics, often resulting in road deformations and cracks under vehicular load [1,2,3]. Traditional replacement methods are resource-intensive, consuming significant amounts of time, labor, and land. Consequently, there is an immediate and compelling need for innovative and sustainable geotechnical solutions for silty soil reinforcement, a topic that is currently the subject of extensive international research. While the use of inorganic additives such as cement, lime fly ash, and lime has been shown to enhance the mechanical and durability properties of silty soils, such treatments often induce brittleness and vulnerability to failure under cyclic or impact loads, leading to various types of cracking [3,4,5,6].

Fiber reinforcement serves as a novel method for soil stabilization, aimed at enhancing geotechnical properties through the uniform integration of various fiber types, including wood, polypropylene, polyester, and polyethylene [7,8,9,10]. This technique notably improves the soil’s shear, tensile, and compressive strengths, as well as its load-bearing capacity. It also increases soil ductility and permeability while reducing the incidence of crack formation and swelling [7,8]. Natural plant fibers such as sisal [11], bamboo, and coir [12] have been widely used for soil reinforcement, and they have effectively improved the engineering performance of soil. Polyester fiber is the largest category of chemical fibers and is the fundamental material for textile and related industries. In 2017, China manufactured 40.8 million tons of polyester, yet the recycling rate stood at less than 8%, contributing to over 35 million tons of waste polyester and subsequent environmental strain [13]. Regenerated polyester fibers (RPFs) stand out for their strength, durability, and cost-effectiveness, making them a suitable choice for stabilizing silty soils. Considering current ecological imperatives, the utilization of RPFs for soil stabilization presents significant economic and environmental advantages [14].

The scholarly community has extensively investigated the mechanical attributes of fiber-reinforced soils, although the preponderance of this research predominantly targets the mechanics of cohesive and granular soils. Investigations by Akbulut et al. [15] and Tang et al. [9] have demonstrated that the tensile strength of fiber-reinforced soft clay can augment by up to 115% with specific fiber concentrations, implying the existence of an optimal fiber content. Studies conducted by Miller et al. [16] ascertain that the optimal fiber concentration for polypropylene fiber-reinforced clay compaction liners ranges between 0.4% and 0.5%, efficaciously mitigating clay layer cracking while bolstering their tensile strength. Amin [17] pointed out that with the increase in fiber length and content in reinforced soil, the energy absorbed by the fibers at the initial crack can effectively reduce crack development. Sujatha [11] employs treated sisal fiber to reinforce the soil; the reinforced soil exhibits the highest strength when the fiber content is 2%. Further work by Shao et al. [18] and Tang et al. [19] on cracking and drying dynamics indicates that fibers are effective in curbing tensile cracking. The research of Balakrishnan et al. [20] corroborates that augmenting fiber length and concentration enhances the soil’s resistance to crack propagation.

Regarding reinforcement mechanisms, Wang et al. [21] employed electron microscopy to discern that fibers forestall additional soil deformation and augment soil mechanical properties. Anagnostopoulos et al. [22] observed that under mechanical stress, fibers and soil particles interact through three primary mechanisms: static friction, rotational resistance, and relative sliding, the latter of which prevails when soil particles exhibit smooth and irregular surfaces. In coarser soils, fibers contribute more significantly to tensile strength, yielding superior reinforcement effects.

In summary, this study utilizes RPFs as the reinforcing material and integrates them with additives such as lime, fly ash, and gypsum powder to enhance the properties of silty soil in Jiangsu, China. Through a comprehensive set of mechanical and physical property tests, the research examines the influence of RPF on post-improvement soil mechanics, analyzes factors affecting these attributes, and evaluates the practicability of employing RPFs for road base improvement in silty terrains. Additionally, preliminary recommendations are provided concerning the optimal length and concentration of RPF for effective soil reinforcement.

## 2. Materials

### 2.1. Experimental Materials

The primary materials employed in this research encompass silty soil targeted for reinforcement, RPFs, and auxiliary substances such as lime, fly ash, and gypsum. Existing literature corroborates that lime and fly ash contribute to elevated compressive strength in soil matrices. Furthermore, lime activates the reactive characteristics of fly ash, thereby amplifying the stability of lime-based compositions. The inclusion of a minor quantity of gypsum functions not only as a setting accelerator but also enhances the cohesion and hardening of the soil mixture that incorporates lime and fly ash, thereby significantly improving its long-term strength.

#### 2.1.1. Silty Soil

**Figure 1:**
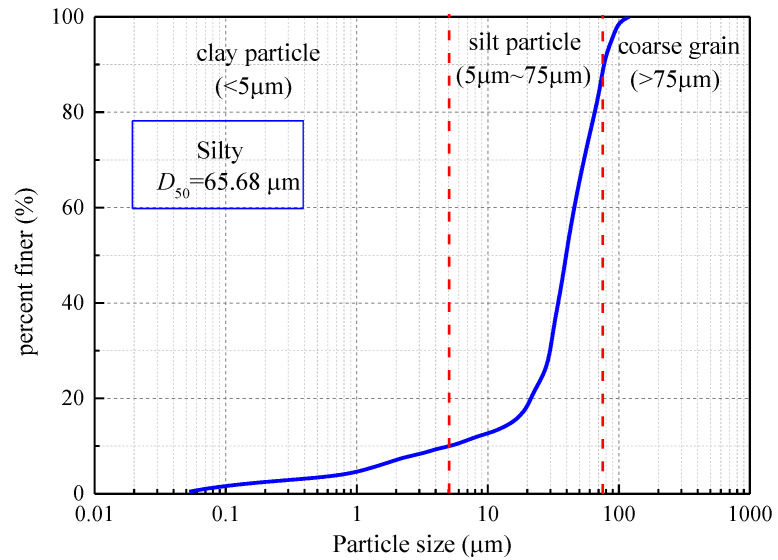
Particle size distribution of the test soil sample.

The silty soil sample for this research was procured from a highway construction locale in Dongtai City, Yancheng, Jiangsu Province, China. Figure 1 delineates the particle size distribution curve of the soil. The sample comprises clay particles with diameters less than 5 μm, constituting 11.3%, silt particles with diameters between 5 μm and 75 μm, accounting for 79.8%, and coarse grains with diameters ranging from 75 μm to 200 μm, making up 8.9%. Table 1 and Table 2 present a comprehensive overview of the soil’s fundamental physical properties and chemical composition. The soil manifests a liquid limit (*w_L_*) below 50 and a plasticity index (*I_P_*) under 10, categorizing it as a low-liquid-limit silty soil in conformity with the Geotechnical Test Procedure (SL 237-1999). The primary constituents include SiO_2_ (61.32%) and Al_2_O_3_ (13.24%), with minor concentrations of CaO, Fe_2_O_3_, and K_2_O.

#### 2.1.2. Recycled Polyester Fiber Properties

**Figure 2:**
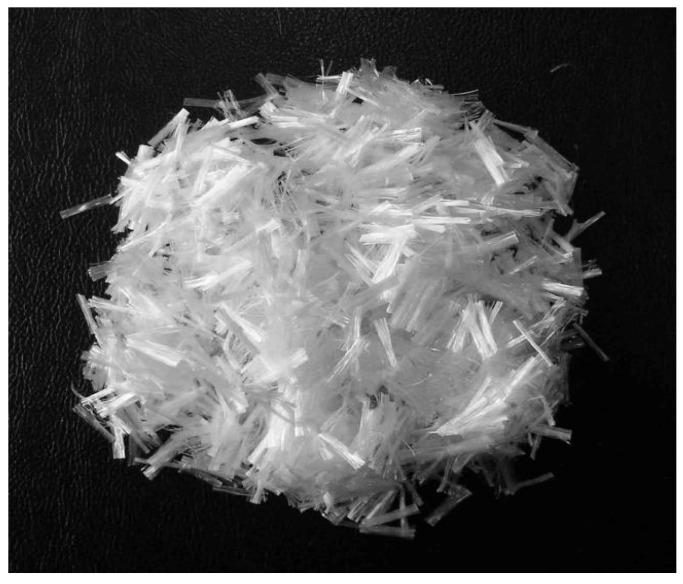
Recycled polyester fiber.

Referred to as RPF, the material is sourced from a specialized textile facility in Tai’an, China. The fiber possesses a density range of 1.31–1.37 g/cm^3^. Regarding tensile strength, the bundled monofilaments demonstrate values ranging from 200 to 400 MPa. The fiber also manifests an elongation at break that varies between 140.6% and 154.7%, as depicted in Figure 2.

#### 2.1.3. Lime, Fly Ash, Gypsum 

Lime is employed as an alkaline activator, serving to establish an alkaline milieu. Table 3 details the essential physical characteristics of the quicklime, while Table 4 provides an in-depth analysis of its chemical composition and concentration levels. Specifically, the combined percentage of CaO and MgO is 65.69%, classifying the substance as Type III quicklime based on its calcium-rich constitution. 

Compared to cement, fly ash presents a cost-efficient alternative and demonstrates superior durability during freeze-thaw cycles when utilized for soil stabilization [23]. Table 5 and Table 6, respectively, list the main physical changes and chemical composition of the fly ash used in this study. As for gypsum, the research makes use of recycled construction gypsum powder, generated through a multistep process involving hydration and setting of the original construction gypsum, followed by further grinding, calcination, and dehydration phases. The primary component of this recycled gypsum is calcium sulfate hemihydrate.

### 2.2. Experimental Approach

The activity of fly ash can be stimulated by lime, while fly ash can improve the structure’s stability. It has been shown that when the mass ratio of lime and fly ash is 1:2, the mechanical properties of soil can be effectively improved [24]. In the present study, predetermined mass ratios of 4% for quicklime and 8% for fly ash are established, calculated as percentages of the additive mass relative to the total mass of the sample. To enhance the early-stage mechanical strength of the soil specimens, an additional 3% of construction-grade gypsum powder (CaSO_4_·1/2H_2_O) is incorporated as an auxiliary material. Extending the academic contributions of Chaduvula et al. [24] and Tang et al. [9], fiber content is varied across a spectrum of concentrations: 0%, 0.1%, 0.2%, 0.3%, 0.5%, and 0.7%. Fiber lengths are also modulated between 6 mm and 17 mm. Table 7 outlines the comprehensive testing protocol devised to assess the physico-mechanical properties of the modified soil. With the exception of tests focusing on limiting moisture content and particle size distribution, all additional experiments are conducted with soil samples maintained at their optimal moisture levels.

### 2.3. Experimental Methodology

To rigorously examine the enhanced physico-mechanical attributes of silty soil reinforced with RPFs, a comprehensive suite of tests was conducted, ranging from moisture content limits to dynamic triaxial analyses.

#### 2.3.1. Consistency Limit Test

Adhering to the guidelines set forth by the “Highway Geotechnical Testing Procedures (JTG E40-2007)” [25], an optoelectronic liquid-plastic limit measurement device was employed. The samples were mixed with fiber, lime, and fly ash, then cured for the required curing period. A cone with a 76 g weight and a 30° angle was used, and for each soil variety, three replicates were prepared. The average cone penetration depth served as the test outcome.

#### 2.3.2. Shrinkage Test

Conforming to the same testing procedures [25], samples were fabricated with dimensions of 100 mm in height and 50 mm in diameter. Vernier calipers were used to meticulously record alterations in the dimensions of the soil samples post-static compaction, throughout standard curing, and under ambient drying conditions.

The term ‘shrinkage characteristics’ [26] refers to the soil’s propensity for volumetric alterations consequent to moisture evaporation. Such volumetric changes, specifically shrinkage deformations, have significant implications for a range of geotechnical phenomena, including soil cracking, slope stability, and foundation bearing capacity [27]. The linear shrinkage rate (*e_sL_*) is quantified using an equation prescribed by the ‘Highway Soil Test Procedures’ (JTG E40-2007) [25].

(1) $$e_{\mathrm{sL}}=\frac{\Delta H}{H_{0}}\times 100$$

where Δ*H* signifies the variation in the diameter of the sample (mm), a positive value indicates a contraction, and a negative value denotes an expansion; *H*_0_ represents the initial diameter of the sample (mm).

#### 2.3.3. Compaction Evaluation

Utilizing a standard SLJ-1 compactor from the Nanjing Soil Instrument Factory (Nanjing, China), we performed compaction tests in compliance with the official guidelines [25]. The falling hammer, weighing 4.5 kg, had a prescribed drop height of 45 cm. The mold was characterized by an internal diameter of 10 cm and a height of 12.7 cm. The compaction energy was precisely calibrated to 2687 kJ/m^3^.

#### 2.3.4. California Bearing Ratio (CBR) Test

The CBR test was conducted in strict compliance with the previously cited procedures [25], utilizing a CBR-2 cylindrical apparatus. The cylinder featured dimensions of 170 mm in height and 152 mm in diameter.

#### 2.3.5. Unconfined Compressive Strength Assessment

Leveraging an YSH-2 unconfined compressive strength device and following the standard protocols [25], tests were performed at an axial strain rate of 1% per minute. The specimen dimensions were precisely 100 mm in height and 50 mm in diameter.

#### 2.3.6. Resilience Modulus Testing

In accordance with the official guidelines cited earlier [25], a Resilience Modulus Instrument (Model HM-1) was employed for conducting the load-unload sequence. The elastic deformation was quantified using a dial gauge, from which the resilience modulus was subsequently calculated.

## 3. Experimental Results and Analysis

### 3.1. Basic Physical Properties

#### 3.1.1. Limiting Moisture Content

**Figure 3:**
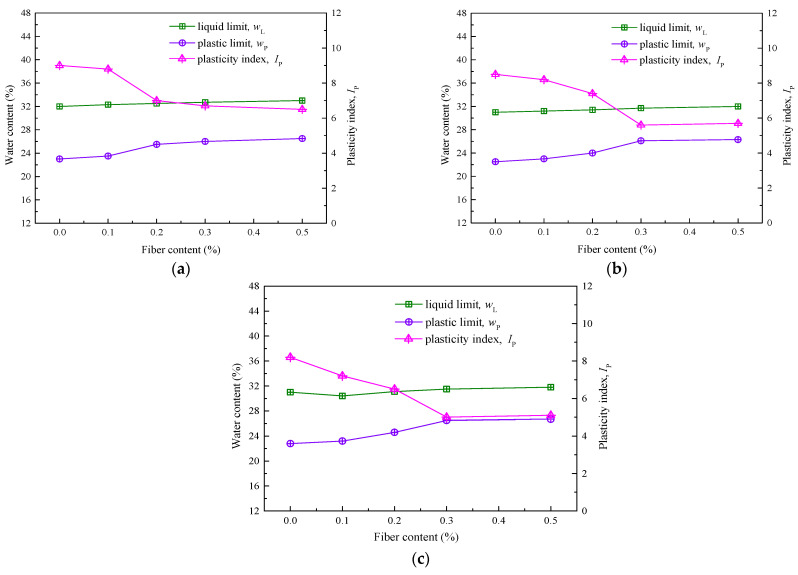
Changes in the boundary water content of RPF-modified silt at different curing ages: (**a**) 1-day; (**b**) 7-day; (**c**) 28-day.

In this study, the liquid limit (*w_L_*) and the plastic limit (*w_P_*) function as diagnostic metrics for evaluating the granular structure and mineralogical composition of soil samples [25]. The plasticity index (*I_p_*), defined as the difference between *w_L_* and *w_P_*, offers valuable information concerning the soil’s plastic state across varying water content levels [28]. Figure 3 graphically represents the temporal evolution of these parameters over 1-day, 7-day, and 28-day curing periods for silty soil fortified with recycled polyester fibers. Under different curing periods, the liquid and plastic limits of reinforced soil slightly increase with the fiber content. The plasticity index experiences minimal variations, oscillating between 2.5 and 3.1. Fibers serve as a form of “soft reinforcement”, optimally functioning when in close contact with soil particles, especially at lower moisture concentrations. Adding fibers cannot improve cohesion but can hold particles together and offer better resistance against load. So whatever change in liquid limit and plastic limit has happened can be attributed to lime or fly ash added to soil, particularly during curing.

Relative to the inclusion of fibers, the curing period exerts a minimal influence on the liquid and plastic limits of the engineered soil. Furthermore, the plasticity index exhibits a diminishing trend over time. Specifically, silty soil enhanced with 0.1% fiber content witnesses a mere 1.6-point reduction in plasticity index after a 28-day curing cycle. Similarly, for soil amended with 0.5% fiber, the plasticity index drops only by 1.4 after the same curing period. The mechanisms influencing the liquid and plastic limits differ between the fiber content and the curing age. While fibers contribute primarily through physical interactions, the aging of the engineered soil triggers intensive hydration reactions. These reactions not only deplete significant water reserves but also generate OH^-^ ions. The copious Ca^2+^ ions, resultant from the hydration of quicklime, partake in ion exchange with soil-bound Na^+^ and K^+^ ions. This process culminates in a thinning of the double layer, coupled with a reduction in bound water content and a continual diminishment of the plasticity index. The emergence of pozzolanic reactions contributes to the formation of cementitious byproducts such as hydrated calcium aluminate and silicate. These substances encapsulate and bond the soil particles, causing an increase in particle size, a corresponding decrease in specific surface area, and a subsequent further reduction in both bound water content and plasticity index [4,26].

#### 3.1.2. Shrinkage Characteristics

**Figure 4:**
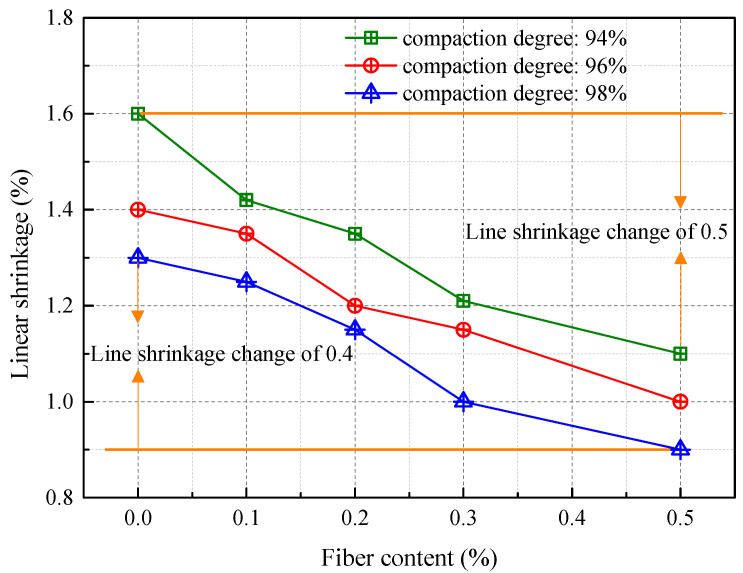
Variations in the linear shrinkage rate of modified soil at different levels of compaction as the fiber content changes.

This section explores the variations in diameter of engineered soil samples compacted to 94%, 96%, and 98% after undergoing 28 days of natural air-drying. Figure 4 visually illustrates a reduction in the soil’s linear shrinkage rate with increasing fiber content. Notably, when soil compaction reaches 96%, the linear shrinkage rate declines significantly from 1.4% to just 1%, supporting the hypothesis that fiber inclusion effectively mitigates shrinkage deformation in engineered soils. Upon completion of the 28-day air-drying period, it becomes apparent that soil devoid of fibers—referred to here as “unmodified soil”—exhibits a faster shrinkage rate compared to soil enhanced with fibers. In particular, the linear shrinkage rate for unmodified soil is roughly 1.04 to 1.45 times higher than that for fiber-augmented soil, underscoring the efficacy of renewable polyester fibers in reducing soil shrinkage. Additionally, increased soil compaction leads to a denser particle structure, which is more effective in minimizing water loss and, by extension, the linear shrinkage rate. Contrastingly, Cheng et al. suggest that, in comparison to pure silty soil, the hydration of lime not only consumes a considerable amount of water but also generates calcium hydroxide (Ca(OH)_2_), filling soil voids [29]. This initially results in a modest volume expansion in the early curing stages, which subsequently contracts as the soil loses moisture. A higher lime content is correlated with increased production of Ca(OH)_2_, thereby yielding less pronounced shrinkage characteristics.

### 3.2. Mechanical Properties

#### 3.2.1. Compaction Characteristics

The moisture content of soil plays a critical role in determining the achievable density of a soil mass. Investigating compaction characteristics and identifying the OMC and maximum dry density are crucial for foundational construction [30]. 

**Figure 5:**
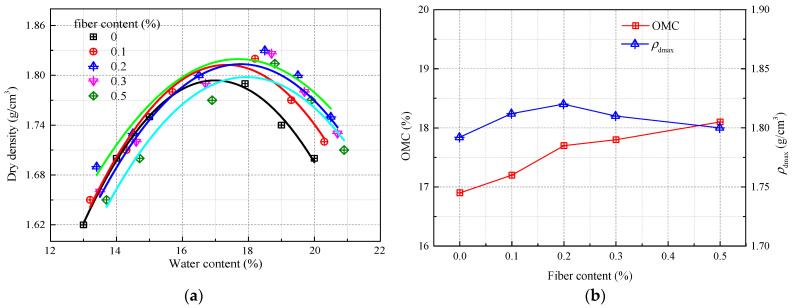
Compaction characteristics of modified soil with different fiber contents: (**a**) compaction curve; (**b**) OMC and *ρ*_dmax_.

**Figure 6:**
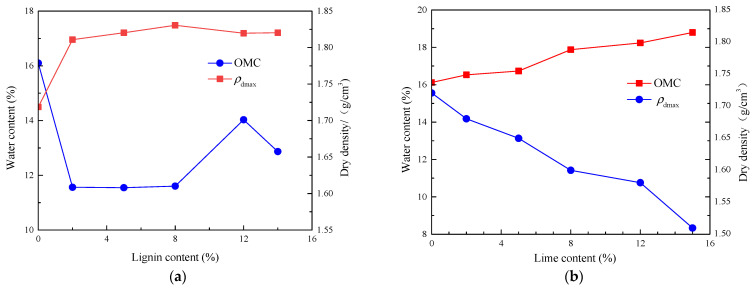
Effect of lignin and lime content on the compaction characteristics of modified silt [4]: (**a**) lignin; (**b**) lime.

Figure 5 shows the compaction curves of modified soil with different fiber contents, with all curves for fiber-modified soil located above those for unmodified soil. Under identical compaction conditions, the OMC of modified soil slowly increases as fiber content rises. Compared to unmodified soil, modified soil with 0.1% fiber content experiences a 9.3% increase in OMC. The maximum density is achieved at a fiber content of 0.2% and is approximately 1.82 g/cm^3^. The density varies only slightly across different fiber contents, with the maximum deviation being 1.56%. The addition of recycled polyester fibers initially increases and then decreases the compaction of the subgrade soil. This suggests that the pores between soil particles are first “tightened” and later “relaxed” as fiber content increases. At optimal fiber content, the fibers form a “dumbbell-like” connection with the fortified soil, maximizing the reinforcing effect of the fibers. When fiber content is excessive, the fibers overlap, reducing both cohesion and friction, leading to a less compact soil mass—a phenomenon referred to as “the looser, the more compacted”. This is different from the effect of adding lignin, which effectively fills the pores between soil particles, as shown in Figure 6 [31]. 

Additionally, Zhang et al. [4,31] pointed out that as lime content increases, the OMC of lime-modified soil also rises, leading to a reduction in density. The mixing of quicklime with soil results in a certain “sandification” effect, enlarging soil particle sizes and increasing inter-particle pores, consequently reducing density. Quicklime also absorbs a significant amount of moisture during the hydration process, generating a large amount of heat and consuming water from the pores, thus leading to an increase in the OMC of the modified soil. Our exploratory tests on different materials for soil modification reveal distinct mechanisms of improvement.

**Figure 7:**
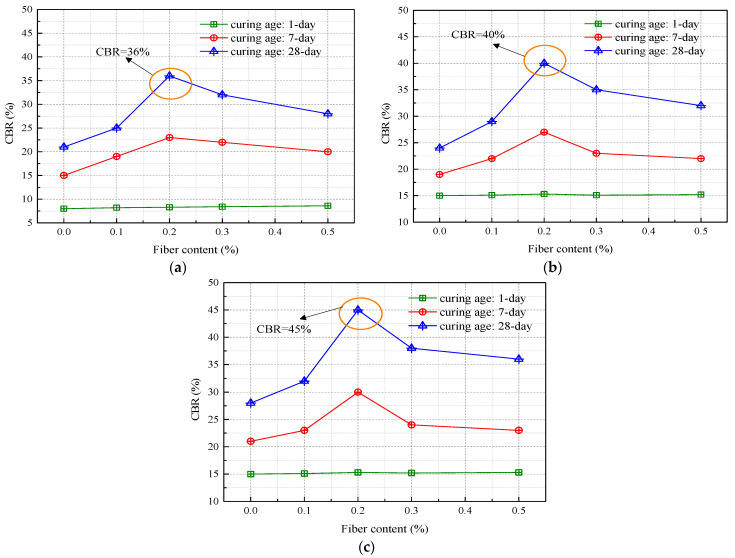
Variations in CBR of recycled polyester fiber-modified soil: (**a**) compaction degree of 94%; (**b**) compaction degree of 96%; (**c**) compaction degree of 98%.

The CBR test serves as a pivotal evaluation method for assessing the inherent mechanical strength of materials deployed in road subgrades, sub-bases, and base courses. This metric functions as a revealing indicator of a material’s resistance to localized shear forces. Figure 7 elucidates how the incorporation of recycled polyester fibers into soil markedly impacts its CBR values under disparate compaction scenarios. According to the criteria set forth in the “Highway Foundation Design Code” (JTG D30-2015) [32], the specific subgrade filler being analyzed fails to satisfy the prescribed minimum CBR requirements. Notably, after a one-day curing period, the CBR values of fiber-augmented soils closely approximate those of their unmodified equivalents. However, during a seven-day curing period, the CBR values exhibit a nuanced increase, reaching an apex at a fiber concentration of 0.2%. Beyond this point, a declining trend in CBR values is observed. At the optimal fiber content of 0.2%, the fibers connect the soil-like bridges, maximizing the reinforcement effect.

**Figure 8:**
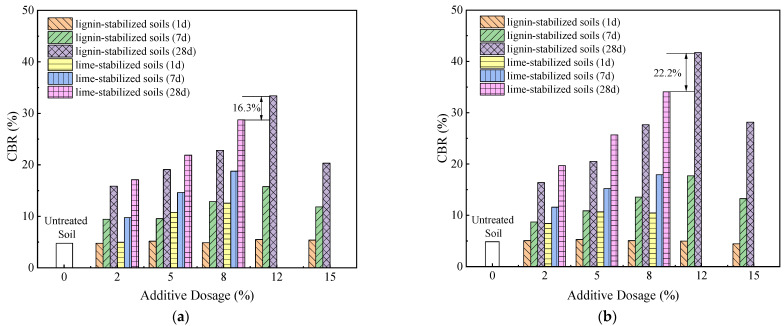
CBR of lignin and lime-modified soil under different compaction degrees [4]: (**a**) compaction degree of 94%; (**b**) compaction degree of 96%.

The curing duration emerges as a significant determinant of CBR values. During the initial seven days of curing, a modest increase in CBR is registered, although it remains below the threshold of 28%. Upon completing a 28-day curing cycle, the CBR values dramatically increase. Specifically, soil fortified with 0.2% fiber content and compacted to 98% yields a CBR value of 45%, thereby meeting the standards for road subgrade filler material. Moreover, complementary research by Zhang et al. on soil modification for road bases using lignin and lime offers additional insights (see Figure 8) [4]. When subjected to identical curing durations, compaction percentages, and additive concentrations, lignin-modified soil consistently underperforms lime-modified soil in CBR metrics. However, this disparity can be ameliorated by increasing the lignin content. Remarkably, soil with a 12% lignin concentration at a 94% compaction rate outperforms its lime-modified counterpart (8% concentration) by 16.3% in CBR values, a lead that extends to 22.2% at a 96% compaction rate [4,31].

#### 3.2.2. Unconfined Compressive Strength

**Figure 9:**
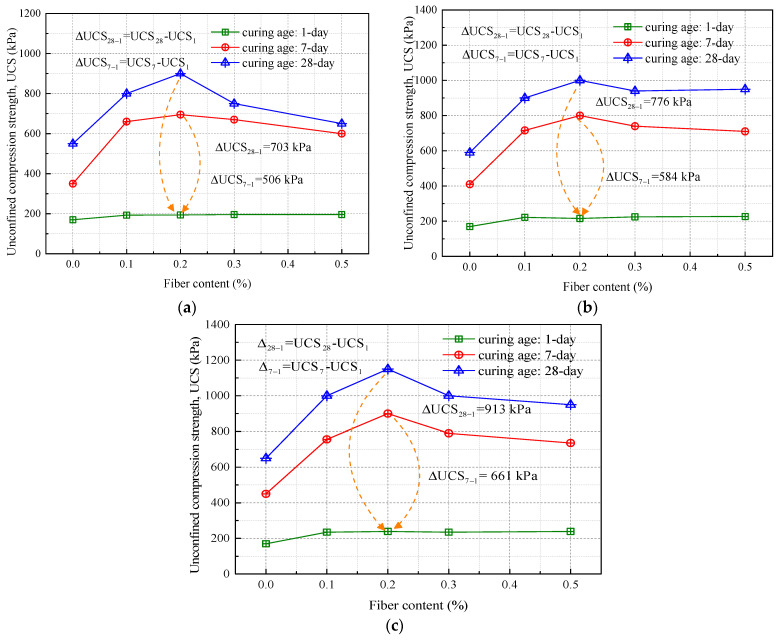
Unconfined compressive strength of improved soil under different compaction degrees: (**a**) compaction degree of 94%; (**b**) compaction degree of 96%; (**c**) compaction degree of 98%.

**Figure 10:**
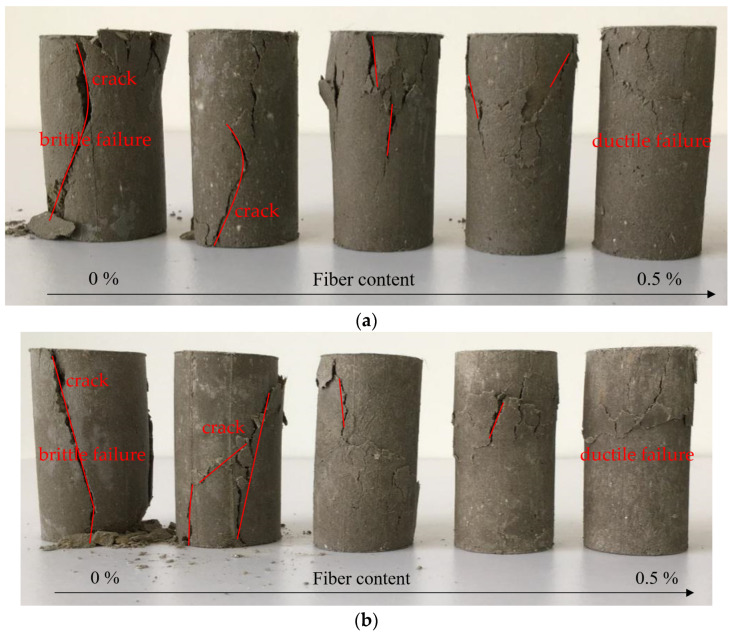
Failure morphology of improved soil samples under an unconfined compression test: (**a**) curing age: 14-days; (**b**) curing age: 28-days.

Figure 9 displays variations in the unconfined compressive strength of soil improved with recycled polyester fibers under compaction levels of 94%, 96%, and 98%. The unconfined compressive strength of the fiber-reinforced soil demonstrates a general increasing trend with the addition of fiber content, peaking at 0.2%. However, beyond this fiber concentration, a decline in unconfined compressive strength is observed. Figure 10 shows the failure morphology of tested soil samples with different fiber contents. As the fiber content increases, a shift from “brittle failure” towards “ductile failure” becomes evident, along with a gradual upward migration of the failure plane. The curing age significantly impacts the strength of the fiber-reinforced soil. After a 1-day curing period, the differences in unconfined compressive strength between soil samples with varying fiber contents are negligible, aligning closely with that of unmodified soil (170 kPa). In contrast, Zhang Tao et al. [4] found that one-day-aged lignin-reinforced subgrade soil exhibited a strength of approximately 100 kPa. 

According to the “Highway Asphalt Pavement Design Code” (JTG D50-2006) [33], semi-rigid base layers are expected to exceed an unconfined compressive strength of 0.6 MPa after a seven-day curing period. At this age, fiber-reinforced soil indeed shows enhanced compressive strength. Specifically, the soil with 0.2% fiber content compacted at 94% achieves a maximum unconfined compressive strength of 695 kPa, well above the specified limit. Moreover, seven-day-aged lignin-reinforced soil (with 12% lignin content) shows a compressive strength of approximately 300 kPa. By the end of the 28-day curing period, the soil undergoes a substantial increase in unconfined compressive strength. For instance, the 0.2% fiber-reinforced soil compacted at 96% exhibits a strength gain of approximately 362%. Tang et al. [9] indicated that fiber-reinforced soil with 0.25% fiber and 8% cement content shows a seven-day unconfined compressive strength about 930 kPa higher than that of soil reinforced solely with cement. Additionally, as the compaction level increases, the variations in unconfined compressive strength between differently reinforced soils widen, reaching maximum discrepancies of 913 kPa and 661 kPa. Research by Mishra et al. [34], Prabakara et al. [35], and Mattone [36] employing different fiber-reinforced clayey soils found that excessively high fiber concentrations lead to a decline in unconfined compressive strength. For subgrade soil in the Jiangsu region, the optimal fiber content for maximizing unconfined compressive strength appears to be around 0.2%.

**Figure 11:**
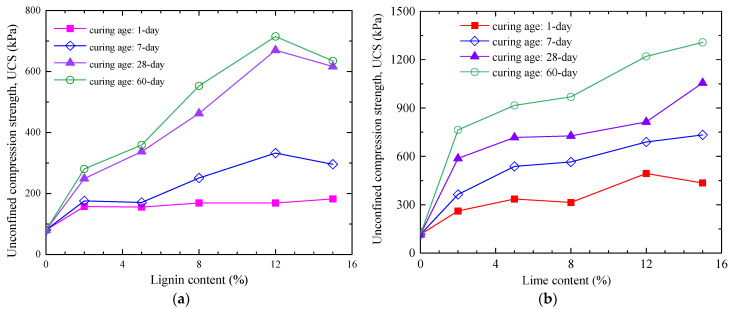
Unconfined compressive strength of lignin and lime-improved soil [4]: (**a**) lignin-modified soil; (**b**) lime-modified soil.

Regarding the issue of unconfined compressive strength (UCS) in road subgrade soil improved with lignin and lime (see Figure 11), Zhang et al. [4] point out: The UCS of lime-improved soil increases with the addition of lime content (ranging from 2% to 15%) and grows as the curing age extends. During a curing age of 0 to 7 days, the UCS of lime-improved soil is higher than that of lignin-improved soil at the same additive content. By the time the curing age reaches 28 days, the UCS of soil improved with 12% lignin is roughly equivalent to that of soil improved with 8% lime [4,31]. However, it is also noticeable that the early-stage UCS of both lignin and lime-improved soils is relatively low. This is mainly because the hydration reactions, pozzolanic reactions, and ionic reactions in the improved soil have not yet fully activated. In contrast, due to the addition of gypsum to the fiber-improved soil, its early-stage strength is higher. At a 98% compaction level, the 7-day UCS of soil improved with 0.2% fiber content can reach approximately 600 kPa.

#### 3.2.3. Resilient Modulus

**Figure 12:**
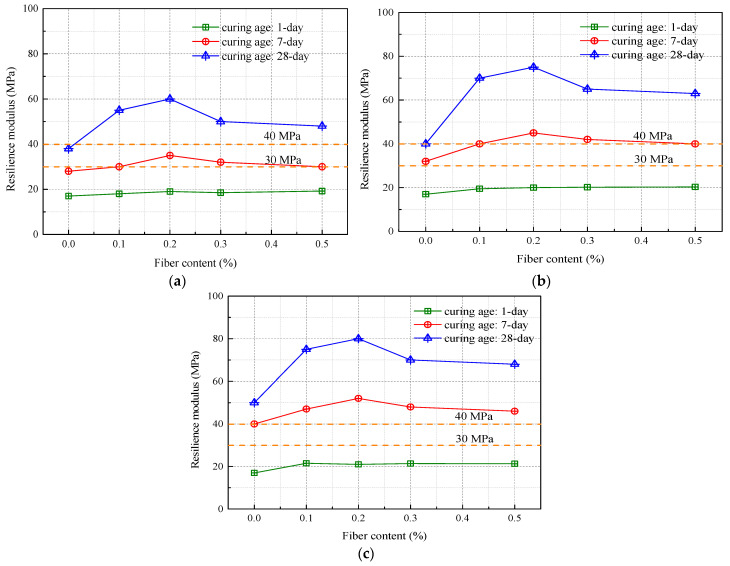
Resilience modulus of fiber-improved soil under different compaction degrees: (**a**) compaction degree of 94%; (**b**) compaction degree of 96%; (**c**) compaction degree of 98%.

The resilient modulus serves as a pivotal metric for gauging both the mechanical attributes and functional performance of roadway foundation materials [37,38]. As illustrated in Figure 12, this modulus undergoes significant enhancements in fiber-augmented soil, particularly as the compaction levels fluctuate. Notably, Zhang et al. [4] observe that at compaction rates of both 94% and 96%, the resilient modulus for subgrade silt soil falls short of the 20 MPa minimum standard established in the “Highway Asphalt Pavement Design Specifications”.

However, the narrative changes substantially when fiber modification is introduced. Within a 7-day curing window, soil samples with a compaction level of 94% meet the regulatory benchmark for highway-grade foundation materials—provided they contain either 0.2% or 0.3% fiber. By day 28 of the curing process, all variations of fiber-enhanced soil conform to these guidelines. This behavior parallels trends seen in other key indicators such as unconfined compressive strength (UCS) and California Bearing Ratio (CBR), where the resilient modulus escalates with incremental fiber content but plateaus once the concentration exceeds 0.2%.

**Figure 13:**
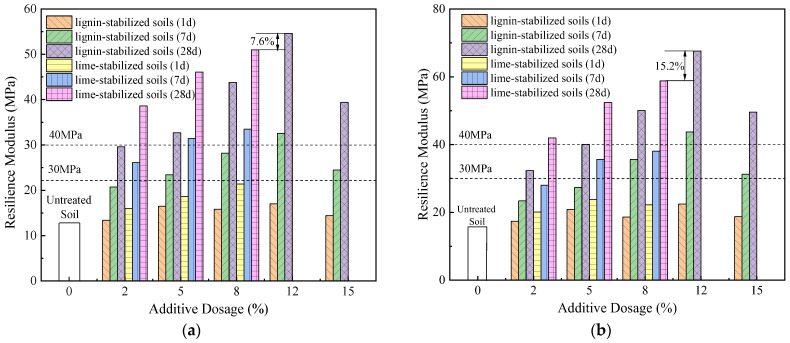
Comparison of rebound modulus of lignin- and lime-modified silt [4]: (**a**) compaction degree of 94%; (**b**) compaction degree of 96%.

In addition, for subgrade silty soils enhanced with lignin or lime (as shown in Figure 13), increasing the lignin content to 12% results in a resilient modulus that is 7.6% (at 94% compaction) and 15.2% (at 96% compaction) higher than soil with 8% lime [31]. On the other hand, the resilient modulus of soil modified with 0.2% fiber content exceeds that of 12% lignin by approximately 7.1% to 15.3%, further confirming that soil improved with 0.2% fiber offers superior performance.

## 4. Analysis of Reinforcement Mechanisms

A comprehensive qualitative and quantitative assessment of variations in porosity, the accumulation of binding materials, and alterations in microstructure before and after the enhancement of silty soil contributes to a deeper understanding of the intrinsic mechanisms behind soil improvement [39,40,41]. Fresh cross-sectional samples (measuring approximately 1 cm^2^) were subjected to electron microscopy after being cured for periods of 7, 14, and 28 days. This analysis allowed for a comparative study of pore-filling and particle-binding characteristics between untreated silty soil and its improved counterpart.

**Figure 14:**
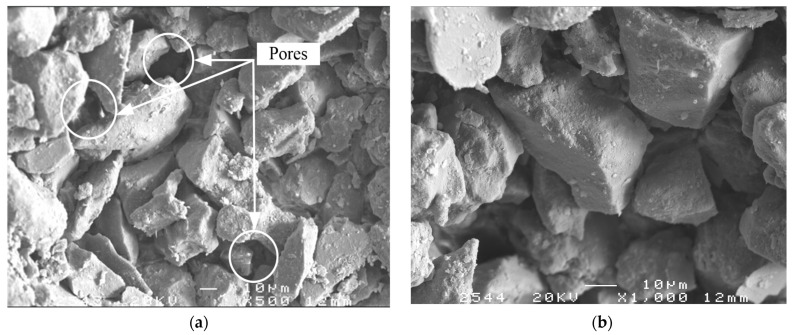
SEM of silt sample (curing age: 28-days) [31]: (**a**) ×500; (**b**) ×1000.

Figure 14 showcases Scanning Electron Microscope (SEM) images comparing unimproved silty soil with soil enhanced through a 28-day curing regimen. In the control samples of native silty soil, micro-particles exhibit a predominantly smooth surface texture. The internal microstructure is characterized by a significant presence of large pores, and the soil particles are defined by distinct edges, irregular peripheries, and acute angular features. Some soil particles even surpass the diameter of these pores.

**Figure 15:**
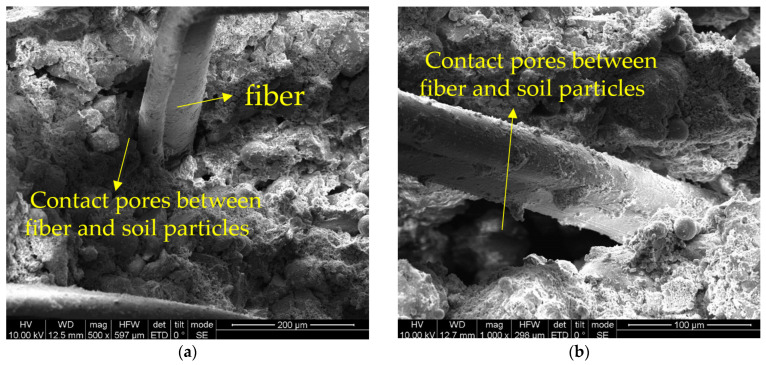
SEM of the modified soil sample (curing age: 7-days): (**a**) ×500; (**b**) ×1000.

Upon introducing specific amendments—namely fly ash, lime, and gypsum—the soil undergoes tangible changes. After a 7-day curing interval, the modified soil still retains a heterogeneous pore distribution. However, a nascent gel-like substance starts manifesting on the particle surfaces and interstices, serving either as a connecting or encasing agent for the soil particles, as visualized in Figure 15. The presence of pores persists at the juncture between fibers and soil particles, suggesting incomplete compaction.

**Figure 16:**
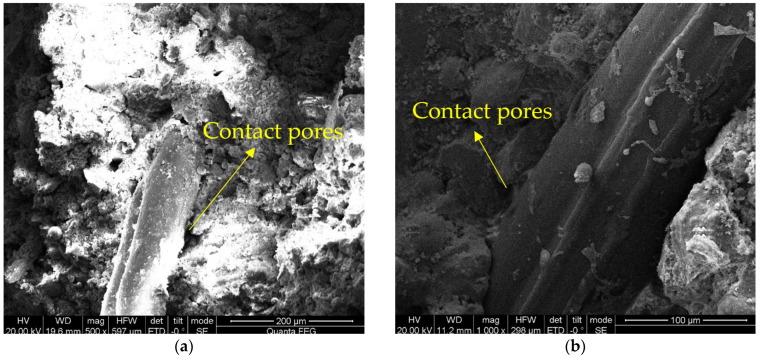
SEM of the modified soil sample (curing age: 14-days): (**a**) ×500; (**b**) ×1000.

**Figure 17:**
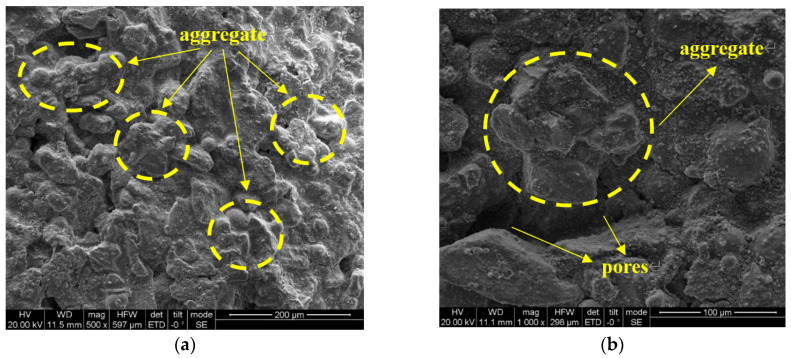
SEM of silt sample (curing age: 28-day) [31]: (**a**) ×500; (**b**) ×1000.

At the 14-day curing milestone, the soil’s microstructure is visibly more compact than its untreated counterpart, with Figure 16 corroborating this claim. Abundant binding materials act as a cohesive agent between soil particles, effectively filling interstitial spaces. Upon reaching the 28-day curing threshold, the encapsulating and interstitial-filling actions of the binding materials are markedly enhanced. Soil particles exhibit closer inter-particle contacts, giving rise to a more stable and densely packed structural configuration, as captured in Figure 17.

Michael J. McCarthy et al. [42] explored the use of fly ash and lime as amendments to improve the durability characteristics of sulphate-bearing soils. The hydration by-products formed from these additives function as encapsulating agents, enveloping individual soil particles and fostering a cohesive, bonded structure. In this synergistic environment, sulphate-bearing soils transition into a more compacted state. The binding substances serve not only to encapsulate the silty soil particles but also, through the mediating action of calcium aluminates [43,44], to significantly increase both the number and diameter of soil aggregates. This, in turn, leads to a reduction in specific surface area while the binding materials fill inter-particle voids, thereby elevating the soil’s compactness.

Consequently, the modified soil experiences an uptick in particle size and maximum dry density, resulting in improved mechanical and roadway performance metrics and a concurrent decrease in the plasticity index. However, there is a cautionary note: when the fiber content in the modified soil exceeds optimal levels, a degradation in mechanical properties is observed. This is potentially attributed to localized fiber clustering and overconcentration, causing slippage at the fiber-soil interface and undermining the structural integrity, thus leading to decreased strength and CBR values. Electron microscopic imagery provides a vivid, direct insight into the microstructural evolution of soil pre-enhancement and post-enhancement, allowing for speculative identification of material modifications [4,42]. To fully quantify these changes in the material composition and microstructure, however, additional analytical assessments are still required.

## 5. Conclusions

This research comprehensively examines the influence of recycled polyester fibers on the physico-mechanical attributes of silty soil and can be applied to the field of improving silt subgrade. The study not only quantifies the impact of variables such as fiber dosage and curing duration on soil properties but also explores the underlying mechanisms that contribute to the soil’s enhanced performance when treated with both recycled polyester fibers and inorganic stabilizers. The salient conclusions are:

(1) Fiber-augmented soil exhibits superior liquid and plastic limit values compared to its non-fiber counterpart, increasing incrementally within a range of 2.58% to 3.12% as fiber content rises. The plasticity index remains relatively stable, fluctuating modestly between 2.5 and 3.1. The role of curing age in these metrics is found to be minimal, while the plasticity index shows a marginally declining trend over time. Remarkably, fibers have a notable dampening effect on soil shrinkage, reducing it by a factor of approximately 1.04 to 1.45.
(2) The incorporation of fibers elevates the OMC and initiates an initial uptick in the maximum dry density (*ρ*_dmax_), which subsequently declines. The peak *ρ*_dmax_ achieves a value of 1.82 g/cm^3^. However, the variance in *ρ*_dmax_ across different fiber concentrations is negligible, with a maximal deviation of around 1.56%. In the fiber concentration window of 0–0.2%, the OMC experiences a slight rise of about 0.8%, while *ρ*_dmax_ marginally escalates. Beyond this range, OMC experiences a slight ascent, whereas *ρ*_dmax_ shows a nominal descent.
(3) Recycled polyester fibers are highly effective in bolstering the roadway applicability of silty subgrade soil. After a curing period of 28 days, the soil surpasses highway subgrade design benchmarks for both the CBR and resilient modulus. Specifically, a CBR value of 45% is attained with a 98% compaction and a 0.2% fiber concentration.
(4) The unconfined compressive strength of the soil improves synergistically with increasing fiber dosage and curing period. However, a decline in strength is observed when the fiber dosage surpasses 0.2%, with the peak strength value approximating 900 kPa. For silty soils native to Jiangsu, an optimal fiber dosage of around 0.2% is recommended.
(5) A microstructural examination discloses the presence of binding substances enveloping soil particles in the treated soil. These substances facilitate the agglomeration of smaller particles into larger clusters while simultaneously filling the interstitial voids to varying degrees, rendering the soil structure more compact and robust compared to untreated soil.

## Figures and Tables

**Table 1:** Main physical property indices of the test soil sample.

Soil Properties	Content
Plastic limit (%)	22.8
Liquid limit (%)	31.6
Plasticity index	8.8
Maximum dry density (g/cm^3^)	1.81
Optimum moisture content (%)	16.45
Specific gravity	2.71
pH	8.21

**Table 2:** Chemical composition analysis results of the soil sample.

**Chemical Composition**	SiO_2_	Al_2_O_3_	CaO	Fe_2_O_3_	K_2_O	MgO	Na_2_O	SO_3_	P_2_O_5_
**Content/%**	61.32	13.24	6.68	3.41	2.61	2.47	2.17	0.23	0.18

**Table 3:** Basic physical attributes of lime.

Specific Gravity	pH	Clay Particle Content/% (<2 μm)	Silt Particle Content/% (2–75 μm)	Coarse Grain Content/% (>75 μm)
3.31	12.4	5.4	42.7	51.9

**Table 4:** Chemical composition and concentration levels of lime.

**Chemical Composition**	CaO	SiO_2_	Al_2_O_3_	Fe_2_O_3_	MgO	SO_3_	Na_2_O	K_2_O	TiO_2_
**Content/%**	65.23	2.62	1.16	0.74	0.46	0.13	0.20	0.18	0.053

**Table 5:** Primary physical properties Indices of the fly ash.

Specific Gravity	Optimum Moisture Content (%)	Maximum Dry Density (g/cm^3^)
2.15	23.2	1.34

**Table 6:** Primary chemical composition of fly ash.

**Chemical Composition**	CaO	Fe_2_O_3_	Al_2_O_3_	SiO_2_
**Content/%**	2.8	7.9	28.4	46.2

**Table 7:** Experimental scheme for assessing physico-mechanical indicators of modified soil.

Test Project	Fiber Content (%)	Fiber Length (mm)	Curing Age (Day)	Other Description
limit water content	0, 0.1, 0.3, 0.5, 0.7, 1.0	6, 9, 12, 15, 17	1, 7, 28	---
shrinkage test	0, 0.1, 0.3, 0.5, 0.7, 1.0	6, 9, 12, 15, 17	7, 28	compaction degree: 94%, 96%
compaction test	0, 0.1, 0.3, 0.5, 0.7, 1.0	6, 9, 12, 15, 17	1	heavy compaction
CBR	0, 0.1, 0.3, 0.5, 0.7, 1.0	6, 9, 12, 15, 17	1, 7, 28	compaction degree: 94%, 96%, 98%
unconfined compression strength	0, 0.1, 0.3, 0.5, 0.7, 1.0	6, 9, 12, 15, 17	1, 7, 28	compaction degree: 94%, 96%, 98%
resilience modulus	0, 0.1, 0.3, 0.5, 0.7, 1.0	6, 9, 12, 15, 17	1, 7, 28	compaction degree: 94%, 96%, 98%
dynamic stress and dynamic modulus	0, 0.1, 0.3, 0.5, 0.7, 1.0	6, 9, 12, 15, 17	1, 7, 28	compaction degree: 94%, 96%

## Data Availability

Not applicable.
